# A randomized controlled trial of a virtual reality based, approach-avoidance training program for alcohol use disorder: a study protocol

**DOI:** 10.1186/s12888-020-02739-1

**Published:** 2020-06-30

**Authors:** Angelina Isabella Mellentin, Anette Søgaard Nielsen, Leonie Ascone, Janina Wirtz, Jerzy Samochowiec, Jolanta Kucharska-Mazur, Friedrich Schadow, Zofia Lebiecka, Tomasz Skoneczny, Nicolai Mistarz, Thomas Bremer, Simone Kühn

**Affiliations:** 1 Unit for Clinical Alcohol Research, Unit for Psychiatric Research, Department of Clinical Research, University of Southern Denmark, J. B. Winsløwsvej 18, 5000 Odense Center, Denmark; 2 Brain Research-Inter-Disciplinary Guided Excellence (BRIDGE), Department of Clinical Research, University of Southern Denmark, Odense C, Denmark; 3 Tele-Psychiatric Center, Region of Southern Denmark, Odense C, Denmark; 4 Neuroplasticity Research Group, Department of Psychiatry and Psychotherapy, University Medical Center Hamburg-Eppendorf, Hamburg, Germany; 5 Pomeranian University of Medicine, Szczecin, Poland; 6 Lise Meitner Group for Environmental Neuroscience, Max Planck Institute for Human Development, Berlin, Germany; 7 Physikalisch-Technische Bundesanstalt Braunschweig, Braunschweig, Germany

**Keywords:** Alcohol use disorder, Approach-avoidance training program, Virtual reality

## Abstract

**Background:**

The approach-avoidance training program (AATP) has shown preliminary promise as an add-on to standard treatment for alcohol dependence. However, knowledge is lacking as to whether the effectiveness of AATP can be enhanced further when performed in a typical drinking situation. The main aim of this study is to investigate whether approach-avoidance training implemented in a virtual reality bar environment is superior to the classical joystick PC-version of the AATP.

**Methods:**

The study will be implemented as a randomized controlled trial. A total of 204consecutively enrolled alcohol use disorder (AUD) patients, recruited from alcohol inpatient clinics in Germany, Poland and Denmark, will be randomized into one of three groups at the start of standard alcohol treatment: group A) stimuli-relevant AATP + treatment as usual (TAU); group B) stimuli-relevant AATP in virtual reality + TAU, and group C) TAU only (control group). Treatment outcomes will be assessed at pre-treatment, post-treatment and 3-month follow-up. Repeated-measures ANOVA will be applied to compare the trajectories of the groups over time on drinking, craving and impulsiveness outcomes. It is hypothesized that the two experimental groups will achieve better treatment outcomes compared to group C and that group B will achieve better outcomes than group A.

**Discussion:**

This study is the first trial examining the effectiveness of stimuli-relevant AATP delivered in a VR environment. The use of VR has shown promise in enhancing the effectiveness of other psychological treatments and since AATP has already been shown effective as add-on treatment, it is of interest to investigate whether these effects can be further enhanced by implementing the program in more ecologically valid environments. If proven effective, the AATP-VR can, like the AATP, be implemented easily and cheaply as add-on treatment or continued care to enhance the effectiveness of current evidence-based treatment.

**Trial registration:**

ClinicalTrials.gov ID: NCT04283305

Registration date: 24.02.20

## Background

Alcohol use disorders (AUD) are associated with cognitive dysfunctions such as cognitive biases in the systems processing alcohol-related stimuli, which may play an important role in the maintenance of addictive behavior and impede the effectiveness of conventional evidence-based treatments [1–3].

Dual process models suggest that addictive behavior is influenced by two semi-independent cognitive systems: (1) a fast and automatic associative “impulsive system” that automatically evaluates stimuli in terms of motivational significance and triggers an approach or avoidance response based on this evaluation, and (2) a slow and controlled “reflective system” that regulates the automatic and implicit responses elicited by the impulsive system based on explicit and controlled higher cognitive processes [4–7]. It has been proposed that the impulsive system in addictive behavior is driven by cognitive biases: alcohol cues automatically capture attention (attentional bias) and elicit an approach action tendency (approach bias) promoting alcohol seeking and drinking behavior [8, 9]. AUD can be conceptualized as a dysfunction in both the impulsive and reflective systems. An over-activated impulsive system is sensitized and triggers cognitive biases towards alcohol cues, while a relatively under-activated and weakened reflective system is unable to regulate these biases. Because the impulsive system is partly automatic and implicit, biases towards alcohol cues in the environment lead to the maintenance of self-destructive drinking behavior despite explicit knowledge about the consequences generated by the reflective system [6, 7, 10].

Conventional evidence-based psychological treatments for AUD such as cognitive-behavioral therapy (CBT) rely mainly on the modification of reflective cognitive processes, while impulsive cognitive dysfunctions are addressed to a lesser extent and typically by applying cue exposure therapy (CET). During CET, patients with AUD are exposed to alcohol to elicit cravings while refraining from habitual approach action tendencies, that is, drinking behavior. With repeated exposure, the sensitization of the impulsive system is assumed to decrease over time. To date, CET has shown limited effects on alcohol consumption outcomes [11]. It has been suggested that this conventional method may not be effective because it fails to provoke cravings and approach biases when delivered in clinical settings, which is a pre-requisite for desensitizing the impulsive system [12, 13].

Novel psychological treatments targeting the impulsive system through the modification of cognitive biases are emerging building on experimental paradigms from clinical neuropsychology [14–16]. One approach targets attentional biases by training attention away from alcoholic cues and towards non-alcoholic cues [14, 15]. Another method targets approach biases by training approach action tendencies towards alcohol to react with avoidance action tendencies [16]. One treatment that has shown preliminary promise in clinical settings is the approach avoidance training program (AATP). This program builds on the diagnostic alcohol-approach-avoidance task (alcohol-AAT) [17, 18], which measures approach bias. During the alcohol- AAT, AUD individuals are requested to react to pictures of alcoholic drinks using avoidance responses (by *pushing* a joystick) and react to pictures of non-alcoholic drinks using approach responses (by *pulling* a joystick). Approach bias is indicated when reaction times are faster for approaching alcohol cues than for avoiding them, whereas the opposite indicates avoidance bias. The AATP is an attempt to modify approach biases for appetitive alcohol cues in sub-clinical and clinical AUD samples [16, 19–21]. During the AATP, patients are typically trained to ‘avoid’ alcohol and therefore requested to react to pictures of alcoholic drinks using avoidance responses (by pushing a joystick) and react to non-alcoholic drinks using approach responses (by pulling a joystick).

In contrast to other novel training programs targeting attentional bias [14, 15] and conventional CET methods [22–24], the AATP is more ‘embodied’ in that patients are requested to actively train action tendencies by reacting with pull vs. push motor responses (e.g. [25]). The AATP has shown promise in decreasing reaction times towards alcohol cues and alcohol consumption in sub-clinical and clinical AUD samples. In an initial experimental trial conducted on a subclinical sample of heavy drinking college students, it was found that those who were trained to avoid alcohol cues drank less in a taste test compared to those who were trained to approach alcohol [16]. In three subsequent clinical trials, AATP was found to be effective in retraining AUD patients to avoid rather than approach alcohol cues, and it was associated with a reduction in relapse rates [8, 19, 20]. Further, a study examining whether AATP could be successfully delivered to a sub-clinical AUD sample via a fully automated web-based delivery pathway also found a reduction in alcohol consumption [26]. In addition, it has recently been shown that AATP may be effective in reducing approach biases even when treating AUD individuals with major and severe alcohol-induced cognitive dysfunctions in the reflective system [27]. Interestingly, functional imaging studies have shown that the intervention decreases brain activity in neural correlates of automatic and implicit cognitive processing (mesolimbic brain regions), suggesting that AATP targets the neural mechanisms behind the maintenance of AUD [9, 21, 25, 28]. Overall, AATP interventions have to date shown better effect in reducing alcohol consumption than conventional methods targeting the impulsive system.

The use of virtual reality (VR) has shown some promise in enhancing the effectiveness of psychological AUD treatments targeting implicit and automatic cognitive processes. In clinical settings, adding VR to CET has been shown to be potentially more effective than conventional delivery methods when it comes to inducing cravings [12, 13]. Although AATP interventions have shown more clinical promise than CET in reducing alcohol consumption, it has been difficult to probe any reductions in craving levels [8, 19, 20, 26]. Adding VR technology simulates and enriches real-life situations by exposing AUD individuals to a wider range of stimuli than possible in clinical settings, which typically only expose AUD individuals to alcohol and not associated situations that can cause cravings and approach responses (e.g. bars and party environments) [13]. If conventional methods targeting the impulsive system can be improved using VR, an emerging question then is whether adding VR to AATP may enhance the effectiveness of this approach.

### Aims

The objectives of the study are to investigate: 1) whether AATP as an add-on to CBT increases the effectiveness of treatment, and 2) whether AATP delivered via VR as an add-on to CBT is superior compared to conventional ATTP.

## Methods/design

The study is registered according to the Standard Protocol Items: Recommendations for Interventional Trials (SPIRIT) guidelines [29] and trial registered: ClinicalTrials.gov ID: NCT04283305, the 24 of February, 2020 and will be conducted based on the CONSORT guidelines [30]. In there are any amendments during the trial, it will be registered on ClinicalTrials.gov.

### Design and setting

The study will be conducted as a multi-national, parallel investigator-blinded randomized controlled trial. Participating countries include Germany, Poland and Denmark.

A total of 204 consecutively enrolled AUD individuals (see Statistical analysis section for the power calculation) will be recruited from inpatient alcohol treatment (68 patients from each country). In each country, participants will be recruited at treatment entry from clinics primarily treating AUD or patients whose primary substance use disorder is AUD. Participants will be recruited from the University Clinic in Hamburg-Eppendorf (Germany), the Pomeranian Medical University Clinic in Szczecin and West Pomerania (Poland), and the inpatient clinic Ringården in Middelfart, Funen (Denmark).

### Treatment as usual at the inpatient alcohol clinics

In all three countries, treatment as usual (TAU) consists mainly of psychological treatment which in some cases is combined with pharmacological treatment in accordance with the NICE guidelines [31]. At treatment entry, the patient is offered detoxification (e.g. diazepam, lorazepam, oxazepam or clomethiazole), and other pharmacological treatment (e.g. acamprosate, naltrexone or disulfiram) is provided to support abstinence if needed. The psychological treatment consists mainly of CBT applied during hour-long individual or group sessions. In Germany, TAU lasts for approximately 1 month, whereas in Poland it lasts for approximately 2 months and Denmark it lasts for approximately 3 months. The inpatient treatment in Germany is shorter due to a shorter coverage of the health insurance. Although patients are encouraged to strive for abstinence, the treatment goal for some patients is moderate drinking, defined as not drinking more than five drinks per occasion. In all countries, the treatment strategy is planned together with the patient and typically incorporates psycho-education, functional analysis of drinking situations, development of coping strategies, problem-solving, and homework between sessions.

The psychological treatments are delivered by trained therapists encompassing nurses, social workers and psychologists. Supervision takes place frequently and psychiatrists and clinical psychologists monitor the treatment course regularly.

### Recruitment

After completing detoxification but prior to starting standard treatment, the patient will be briefly informed about the project and asked if he/she is willing to meet with a research assistant who will provide further information. If the patient agrees, the research assistant will provide him/her with written and oral information about the study. After obtaining informed consent, a baseline interview will be carried out. Patients fulfilling the eligibility criteria will be randomized to one of the three aftercare treatment groups described below.

### Randomization

Randomization will occur using computerized randomization with random numbers. To ensure adequate allocation concealment, the random allocation sequence was 1.) generated before patient enrollment begin, and 2.) by a member in the research group (LA) that is not involved personally with the patients. That is, is not part of the clinical setting were the study is implemented.

### Eligibility criteria

To be eligible to participate, patients must: 1) agree to participate in the study and sign written informed consent; 2) be aged between 18 and 65 years; 3) speak the language of the participating country (German, Polish or Danish); 4) have completed detoxification (if needed); 5) have been enrolled within 2 weeks of standard treatment; 6) not have any sensory or motor deficits complicating the provision of AATP (e.g. color-blindness, fine or gross motor deficits in upper extremities); 7) not fulfil diagnostic criteria for other substance use disorders; and 8) not have a severe psychiatric or neurological illness (e.g. psychotic disorders, mental retardation, dementia) or terminal somatic illness.

### Experimental and control groups

A total of 204 (68 patients from each country) fulfilling the eligibility criteria will be randomized into one of three groups: (A) AATP + TAU (*n* = 68); (B) VR-based AATP + TAU (*n* = 68), and (C) TAU (*n* = 68). The mean optimal effect of AATP among AUD patients is typically achieved after the sixths training session [32]. Therefore, group A and group B will receive six sessions (three sessions per week for 2 weeks; duration = 30 mins) of AATP and VR-based AATP, respectively, in addition to TAU. Group C will only receive TAU. The two experimental groups will begin treatment approximately 3 weeks before discharge from the inpatient clinics to measure the add-on effect and to ensure that the add-on treatment does not extend the treatment period.

#### Conventional approach- avoidance training program

Since the AATP builds on the experimental neuropsychological paradigm, the training is intertwined with the alcohol-AAT and consists of a pre-test, training, and post-test session.

In the AAT, patients are instructed to respond to either the *content* or the *format* of the presented pictures using a joystick. When responding to the content in the “stimuli-relevant” trials, they have to pull the joystick to approach pictures of alcoholic or non-alcoholic drinks and push the joystick to avoid the pictures. When responding to the format in the “stimuli-irrelevant” version, they must pull or push the joystick based on the color of the frame around the pictures of the alcoholic or non-alcoholic drinks. The color of the frame is interchangeably reversed (through randomization): a yellow frame requires a push response and a blue frame requires a pull response. Pulling the joystick increases the size of the pictures and pushing it decreases the size, generating a perception of approach and avoidance, respectively.

The pre-test for the AAT starts with practice trials in which patients learn to push and pull the joystick. Actual pre-test trials follow, with patients completing either the stimuli-irrelevant AAT or the stimuli-relevant version first in random order. In both versions, the pictures of alcoholic and non-alcoholic drinks are presented equally often in push and pull format. Following this, patients are requested to complete the avoid-alcohol training in the stimuli-relevant AATP. That is, during the training the patients are *only* instructed to respond to the *content* of the stimuli. In the training trials, 100% of the alcohol pictures come in push-format and 0% in pull-format, with reversed contingencies for pictures of non-alcoholic drinks. Half of the pictures used in the pre-test will also be applied in the training, and the half will be different to allow a test for generalization to untrained pictures. The post-test for the stimuli-irrelevant/stimuli-relevant AAT is almost identical to the pre-test: it presents both trained and untrained pictures of alcoholic (50%) and non-alcoholic (50%) drinks [19] (see Supplementary material for further description of the parameters [33]).

Before and after each session, patients will be requested to report their immediate level of cravings on a VAS scale (see Secondary outcome: cravings, self-efficacy, impulsivity and depressive severity and Cue-induced cravings sections for further details).

#### Approach- avoidance training program in virtual reality

In the VR-based AAT and AATP, patients are situated in a bar environment where drinks appear on a table in front of them. Similar to conventional AAT and AATP (delivered on a computer), the VR-based AATP consists of a pre-test, training, and post-test session, but patients are requested to respond to either the content or format of the presented beverages by pulling or pushing a VR controller instead of a joystick.

The pre-test for the VR-based AAT starts with practice trials in which patients learn to use the VR controller. This is followed by actual pre-test trials: patients complete the stimuli-irrelevant AAT first followed by the stimuli-relevant AAT. In the stimuli-irrelevant AAT, there is a colored outline around the beverages which patients must respond to. The colored outline is interchangeable reversed (via randomization), with a yellow outline requiring a push response and a blue outline requiring a pull response. In the stimuli-relevant AAT, patients must respond to the content of the bottles. Both versions present alcoholic and non-alcoholic drinks equally often in push and pull format. Patients are then requested to complete the avoid-alcohol training and respond to the *content* in the stimuli-relevant VR-based AATP. In the training trials, 100% of the alcoholic beverages come in push-format and 0% in pull-format. The contingencies are reversed for non-alcoholic drinks. Half of the beverages used in the pre-test will also be applied in the training, and the other half will be different to allow a test for generalization to untrained alcoholic. Similar to the conventional ATT, the post-test for the stimuli-irrelevant/stimuli-relevant VR-based ATT is almost identical to the pre-test.

In the VR-based AATP, pulling the VR controller will approximate the drink (will bring the drink closer) and pushing it will distance the drink, which we anticipate generates a more real perception of approach and avoidance, respectively, relative to the zooming feature in the conventional AATP. Further, the VR-based AATP will prompt patients to exercise more realistic movements compared to the conventional AATP, and the virtual bar context may make the setting appear more like real life. Also, a bar tender will be placing the alcoholic beverages onto the bar counter, which may enhance the amount of effort used to avoid and push drinks away. Patients will be seated on a bar stool in VR, and the alcoholic and non-alcoholic beverages will be depicted as high-poly objects to make the experience as realistic as possible (see Supplementary material for further description of the parameters [33]).

Similar to the conventional AATP, patients will be asked to rate state craving on a VAS scale before and after training.

#### Treatment as usual

The control group will not receive any add-on sham-training since prior studies testing the AATP did not find any difference between AATP-sham-training and no-training [19]. Patients in this group will be randomly assigned to complete the stimuli-relevant and stimuli-irrelevant AAT on either the PC- or VR-based bias assessment and will only be assessed at baseline and post-treatment (see Measures section). The conventional and VR-based AATP will be delivered by a trained research assistant.

### Measures

**Fig. 1:**
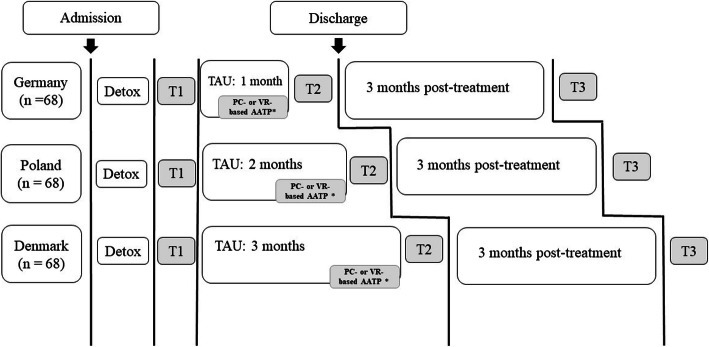
Overview of the study design across assessment sites (countries). Abbreviations: AATP: approach-avoidance training programme; PC: personal computer; T1: baseline assessment; T2: post-treatment assessment; T3: 3month follow-up; VR: virtual reality. * Both add-on experimental AATP interventions starts 3 weeks before discharge

Socio-demographic factors, AUD diagnoses and severity, prior AUD treatment, psychiatric comorbidity, medication, behavioral inhibition and treatment goal will be assessed in the baseline interview (T1). Clinical outcome measures relating to alcohol consumption and cravings will be assessed at baseline, post-treatment, and 3-month follow-up (T1, T2, and T3). Additionally, experimental outcomes, including approach biases, cue-induced cravings and a response inhibition task, will be assessed at baseline and post-treatment (T1 and T2) (See Fig. 1 for an overview of the assessment and intervention timeline across countries). Finally, the patients will be assessed for concomitant treatment between the post-treatment and 3-month follow-up at the 3-month follow-up assessment (T3). The assessments will, like the interventions, be carried out by trained research assistants.

#### Baseline assessment instruments

The Mini-International Neuro-psychiatric Interview (MINI) for DSM-5 is a structured interview probing the 17 most common psychiatric diagnoses using dichotomous questions that ask for a yes/no response. The MINI will be used to establish the severity of AUD diagnoses as well as to screen patients for eligibility and detect psychiatric comorbidity diagnoses [34, 35].

The European version of the Addiction Severity Index (EUROP-ASI) is a semi-structured interview assessing sociodemographic variables as well as AUD severity and seven potential problem areas (alcohol use, drugs use, medical problems, psychiatric problems, family problems, employment/economy problems, and legal problems) [36, 37]. The EUROP-ASI will be applied to standardize the assessment of sociodemographic data and AUD severity across all sites.

#### Clinical outcome measures

##### Primary outcome: alcohol consumption

Alcohol consumption measures will be derived from the Timeline Followback (TLFB). The TLFB method involves using a calendar to identify alcohol consumption patterns in terms of number of drinks per day during the last 30 days [38, 39]. One drink is defined as 12 g of ethanol.

##### Secondary outcome: cravings, self-efficacy, impulsivity and depressive severity

The Visual Analogue Scale (VAS) comprises single-items used to measure the degree of alcohol cravings on scales ranging from 0 to 100, with 0 representing no cravings and 100 an extreme degree of cravings. The scale is presented visually on a ruler, and the individual will be requested to report the mean level and the peak level of cravings experienced during the last 30 days [40, 41].

The 14-item obsessive-compulsive drinking scale (OCDS) measures the degree of cravings experienced during the last 7 days. Items are rated on scales ranging from 0 to 4. The higher the score, the more pronounced the cravings. The scale has two subscales: obsessive cravings score and compulsive cravings score. A total score is calculated based on these subscales, and it is possible to calculate a resistance/impairment and interference subscale score [42].

The 40-item Alcohol Abstinence Self-Efficacy Scale (AASE) measures current level of temptation to drink alcohol and self-efficacy to abstain from drinking. The scale applies 20 situations representing typical drinking cues. Twenty items pertain to temptation levels, the other 20 items to self-efficacy. Items are rated on scales ranging from not at all (0) to extremely (4). The measure comprises the following sub-scales: (1) negative affect; (2) social interaction and positive states; (3) physical and other concerns; and (4) withdrawal and urges [43].

The 30-item Barratt impulsiveness scale (BIS, Patton, Stanford, & Barratt, 1995) assesses dimensions of impulsivity using three subscales: (1) attentional (attention and cognitive instability), (2) motor (motor and perseverance); and (3) non-planning (self-control and cognitive complexity). Items are rated on 4-point Likert scales ranging from 1 = very true for me to 4 = very false for me [44].

The Beck Depression Inventory-II (BDI-II; Beck et a., 1996) is a 21-item multiple-choice instrument that measures the severity of depression. Each item is rated on a 4-point scale ranging from 0 to 3 based on severity [45].

#### Experimental outcomes

##### AAT and diagnostic-AAT-VR

The conventional AAT will be applied as described in Conventional approach- avoidance training program section. The application of the VR-based AATP will be described in Approach- avoidance training program in virtual reality section. The TAU group will be randomized to either the PC or VR AAT bias assessments as described in Treatment as usual. The stimuli in the AAT and AATP stimuli consist in known brands or stimuli that are easily recognized (see Supplementary material for further description and pictures of the stimuli [33]).

##### Cue-induced cravings

Cravings will be assessed for all alcoholic and non-alcoholic beverages from the stimuli-relevant conventional and VR-based AATP. The pictures will be rated on the VAS scale ranging from 0 to 100 according to the immediate level of craving induced to examine their potential to elicit cue-induced cravings.^1^

##### Response inhibition

Response inhibition towards alcohol cues will be recorded using a modified version of the classical Go/No-Go Task [46, 47]. The modification involves the use of alcohol-related and neutral pictures to specifically test inhibition capacities towards alcohol-related cues. Pictures of alcoholic and non-alcoholic drinks from the conventional and VR-based AAT as well as new pictures will be included in the task. Patients will be instructed to respond as fast as possible, and without errors, by pressing a response button when they see a non-alcoholic drink (i.e. “Go” signals) but to withhold their response when an alcoholic drink is presented (i.e. “NoGo” signals) (see Supplementary material for further description of the parameters [33]).

### Data management

All data collected in the study will be treated as strictly confidential and the data collected by research assistants will be entered in a secure database. Before extraction or transferring data across sites, data will be anonymized (person sensitive data) and encrypted (group allocation) by an external data manager and transmitted through secure pathways. Researchers responsible for reporting the results will not have access to the data and will receive the analysis in their final form for reporting. All procedures are approved by the research ethics committee in each of the participating countries (see Ethics section).

### Statistical analysis

Descriptive statistics will be calculated to summarize socio-demographic factors, AUD diagnosis and psychiatric comorbidity as well as primary and secondary measures at baseline. Categorical variables will be described using frequencies and percentages. Normally distributed numeric variables will be described using the mean/standard deviation (SD), non-normally distributed numerical variables using the median/interquartile range (IQR).

The main analyses will be conducted with a repeated measures ANOVA, with group as the between-subjects factor and drinks per day the last 30 days at each assessment time-point as the within-subjects factor, to test the effectiveness of the intervention across the assessment points, which would be indicated by a significant Time X Study Group interaction. If there is an overall significant interaction effect between any one time-point and group, we will examine whether the change over time differs between the groups and the time-point using contrasts. Secondary analysis of continuous outcomes will be conducted with repeated measures ANOVA models, with group as the between-subjects factor and other alcohol consumption- (drinking days and drinks per drinking day), craving-, impulsivity- and depressive symptom outcomes as the within-subjects factor. In the case of unbalanced attrition in the randomization groups, mixed models will be applied instead of repeated measures ANOVA. Secondary analysis of dichotomous outcomes (e.g. relapse) will be conducted using mixed model logistic regressions. The analyses will be adjusted for the randomization stratification variable, site, as well as for the outcome values at baseline [48]. Since the study is a randomized controlled trial, other covariates are expected to be balanced across the randomization groups. Missing data will be handled using multiple imputation with changed equation (MICE) methods applying the non-parametric predicted mean matching (PMM) methods [49].

All analyses will be conducted as intention to treat as well as per protocol, and subgroup analyses will be conducted on patients who were successfully treated with conventional or VR-based AATP, defined as completing all six sessions. Further, moderation analyses of approach bias and using anti-craving medication on the treatment effect will be conducted for the primary and secondary outcomes.

When performing the descriptive and inferential analysis, the researchers will be blinded to the experimental and control groups, and an external statistician will conduct the analysis. The significance level in the models will be set at 5%, and two-sided analyses will be conducted. Effect sizes will be reported in accordance with the statistical modelling. All analyses will be conducted using Stata 16.

#### Power analysis

The present study is the first to focus exclusively on stimuli-relevant AATP training in addition to TAU in clinical AUD patients, and only one prior study has examined the stimuli-relevant AATP training [26]. This prior study was conducted among subclinical AUD individuals and used a continuous drinking (drinks per day) measure as an evaluation of a stand-alone stimuli-relevant AATP over the web. A small effect at post-treatment (f = 0.17) and close to a medium effect at 3-month follow-up (f = 0.22) was identified [26]. Since our study is targeting a clinical sample with a higher degree of alcohol consumption, it may be easier to detect an effect than in a subclinical AUD sample, however, the AATP training in our study is provided as an add-on to TAU which could dilute the treatment effect compared to the prior study. To be conservative, we only aim to achieve a small additive effect at 3-months follow-up. Since pre- and post-treatment assessments of our intervention are conducted in an inpatient setting rather than over the web, attrition is mainly an issue at the 3-months follow-up assessment. Therefore, we collect only the clinical outcomes that can be measured in a variety of settings (e.g. at treatment, at home, over the phone or through a link on the web). An attrition rate of 30% at 3-month follow-up is assumed.

Based on this, a power analysis (PA) [50, 51] was conducted using G*Power for a repeated-measures ANOVA with α = 0.05; effect size (ES) of f = 0.17; power = 0.80; groups k = 3; time-points measurements = 3; correlations among repeated measures = 0.40; and nonsphericity correction E = 0.60, resulting in a sample size of *n* = 123 participants. To allow for adjustment of the stratification variable, site, an extra 20 patients are required for the final analysis (total *n* = 143). Finally, to account for an attrition rate of 30%, the required sample size is 204 (= 143/(1–0.3)) patients, corresponding to 68 patients in each group.

## Ethics

The patients in this study will receive standard treatment at the inpatient clinics and although 66% will receive additional treatment, all patients will be well-treated with evidence-based AUD treatment. Thus, we find no ethical problems with not offering the intervention to the entire sample. However, a critical ethical question is whether using the AATP may encourage patients in the experimental groups to consume alcohol instead of discouraging it. This concern relates to the fact that AAT and AATP involve in vivo exposure to alcohol. However, in European culture, all patients will be exposed to alcohol, both during and after treatment, and they will be unable to avoid this since large-scale alcohol advertisements are on display in the public space, in magazines and on television. Also, alcohol is available, highly visible and easy to buy around the clock in all supermarkets, delicatessens, kiosks and gas stations. Therefore, we consider exposure to alcohol pictures in the experimental groups to be no riskier for the patients than their exposure in everyday life. On the contrary, in the experimental groups, the patients will be trained to avoid alcohol cues and to approach non-alcoholic drinks when exposed in real life. Another ethical question worth considering is whether the VR-based AATP may cause discomfort such as motion sickness (dizziness, nausea, dry eyes etc.). However, patients will not be moving freely around in the VR, which is what usually causes motion sickness. Moreover, the treatment will be performed by healthcare professionals and if any discomfort is experienced, the treatment will be interrupted, and the discomfort alleviated before the end of the training session. Further, we expect the benefits of the VR training to outweigh potential episodes of minor discomfort.

The study protocol has been approved by the research ethics committee in each of the participating countries. In Germany by the local psychological ethical committee at University Medical Center Hamburg-Eppendorf (project-ID: LPEK-0088. In Poland by the Ethical Committee at Pomeranian Medical University in Szczecin (project-ID: KB-0012/162/19). In Denmark by the Regional Scientific Ethical Committees for Southern Denmark (project-ID: S-20190136).

## Discussion

The main aim of this study is to investigate whether stimuli-relevant AATP as an add-on increases the effectiveness of standard treatment and whether stimuli-relevant VR-based AATP is superior compared to the classical joystick PC-version of the AATP.

It is important to emphasize that both the conventional AAT and AATP can be delivered in a stimuli-relevant” and “stimuli-irrelevant” version. In the former version, patients are instructed to focus on the content of the picture (e.g. approach soda, avoid alcohol), whereas in the latter version they are instructed to focus on the format of the picture (e.g. approach horizontal, avoid vertical). Hence, the main difference between the two versions has to do with where the patients’ attention is directed, that is, towards either picture content or an image-irrelevant feature like the picture format. The majority of AAT studies examining AUD and other substance use disorder samples have applied the SI version, even though a recent meta-analysis showed that the stimuli-relevant AAT produces much stronger approach bias measures than the stimuli-irrelevant AAT [52]. Further, alcohol consumption has been found to correlate positively with automatic approach biases in an AUD sample – but only in the stimuli-relevant AAT – suggesting that the stimuli-irrelevant AAT is not a reliable and valid measure of action tendencies in AUD samples [53].

In line with research applying the conventional AAT, only two trials so far have compared the stimuli-relevant and stimuli-irrelevant versions of the AATP in AUD samples [19, 26], whereas other studies have focused exclusively on the stimuli-irrelevant AATP [8, 16, 20]. The first study found the stimuli-relevant AATP to be equivalent to the stimuli-irrelevant version in increasing abstinence rates when delivered as add-on treatment in a clinical AUD sample [19]. The second study demonstrated a superior effect of stimuli-relevant AATP on alcohol consumption compared to the stimuli-irrelevant version in a sub-clinical community sample of AUD individuals [26]. It should be highlighted that none of the AATP studies targeting alcohol approach bias have applied a stimuli-relevant AAT, and this upcoming study is the first to examine whether both stimuli-related and stimuli-irrelevant AAT can be modified by a stimuli-relevant AATP and whether they moderate the effect on clinical outcomes. Studies investigating the effect of AATP on other addictive behavior than AUD have exclusively applied the stimuli-irrelevant version [54–56], which is why it is not possible to draw any inferences from this parallel line of research.

Since the stimuli-relevant AAT and AATP have shown promise in AUD samples, this approach seems particularly suitable for implementation in a VR format, which may be more challenging when directing attention towards image-irrelevant features in natural drinking environments without interfering with its authenticity. To date, VR has primarily been used in attempts to enhance the effectiveness of CET interventions though the induction of cravings. Traditional methods of inducing cravings rely on presenting alcohol in vivo either by placing real alcohol or pictures and videos of alcohol in front of the patient. Although these cues have been shown to induce cravings in some studies [57], and cravings have been shown to predict relapses among AUD patients [58, 59], it has been more difficult to demonstrate a simultaneous decrease in cravings and alcohol consumption in CET trials [11]. Therefore, it has been suggested that, overall, CET interventions may have limited effectiveness because they fail to induce cravings in clinical settings, which is a prerequisite for desensitizing the impulsive system [12, 13]. It has been suggested that integrating alcohol and associated cues in natural drinking environments (e.g. bars, party’s) may be more relevant to induce more cravings than CET, and that VR-based CET may then represent an improvement to CET in that AUD individuals can be taken out of the laboratory or clinical context and placed in an environment with the capacity to induce greater cravings, which in turn might prompt the generalization of treatment to real world activities [12, 60–62].

In sub-clinical and clinical AUD samples, a number of studies have applied VR as an assessment tool to explore alcohol cravings and have found it highly effective in inducing cravings [63–67]. Furthermore, studies examining other drugs of abuse have found that virtual conditions induced more cravings than pictures (e.g. [68–70]. Also, some studies have examined VR-based CET for reducing alcohol-related cravings in AUD samples and found the intervention to be effective in reducing cravings for alcohol [12, 71–73]. The results of assessment and intervention studies are relatively consistent and indicate that if induction of cravings in more alcoholic environments is a major challenge, VR-based CET shows promise for enhancing the treatment of AUD [13].

Cognitive biases and cravings should share a reciprocal relationship, where increases in cravings will make alcohol and associated cues more salient and as these cues become the focus of attention and activate action tendencies they will elicit further increases in subjective craving [74, 75]. Moreover, the association between craving and attentional bias appears to be larger when the strength of craving is relatively high than when it is low at the time of assessment [75], and although this remains to be investigated among substance (ab) users, similar findings have been found between food-craving and approach bias among binge eaters [76].

AATP interventions have to date somehow shown more promise than conventional CET in reducing alcohol consumption but, like with CET, it has been more difficult to prove simultaneous reductions in craving levels and alcohol consumption [8, 19–21]. However, since AAT and AATP also involve in vivo exposure through alcohol pictures, it is plausible that these cues in drinking-related environments may also induce a higher cravings level and cognitive bias, which in turn may improve alcohol consumption measures. In addition to being easier to implement in VR, the stimuli-relevant AATP may have the potential to induce more cravings than the stimuli-irrelevant version because the patients are, like in CET, requested to focus attention on alcohol and associated stimuli [52, 53]. Indeed, it has been suggested that the stimuli-irrelevant AATP may in some cases fail to induce bias because patients totally ignore the content of the picture and only respond to the stimuli-irrelevant content (the format of the picture) due to limits in attention span [77–79]. While assessment tools and interventions targeting attentional biases have exclusively applied stimuli-irrelevant versions (e.g. [14, 15]), in sub-clinical and clinical samples suffering from a wide range of psychiatric disorders, those targeting approach biases can be used in both stimuli-irrelevant and stimuli-relevant versions making them more suitable for VR, where stimuli-relevant versions may be better fitted. However, in this context it is of relevance to notice that a few studies have applied alternate versions of attentional bias modification programs with stimuli-relevant instructions in non-clinical samples to train attentional negative emotional and affective bias. These studies have consistently shown greater or a similar reduction of attentional bias compared to traditional stimuli-irrelevant versions [80–82], which suggests that cognitive bias re-training programs might in general show promise in content relevant versions and be easily implementable in VR formats in the future. Although AATP is already regarded as the most embodied of the cognitive bias re-training programs, the VR format has the potential to make the paradigm even more so. This is because patients are not just requested to actively train action tendencies by reacting with pull vs. push motor responses, but they can navigate in a relatively interactive environment and approach or avoid the alcohol and related stimuli in a wider sense.

In the realm of the available evidence from prior AATP studies it is hypothesized that the two experimental groups will achieve better treatment outcomes (i.e. significantly lower drinking, cravings and impulsivity) compared to the control group, and that the VR-based AATP will achieve better outcomes than conventional AATP at tree month follow-up. The current study will provide important knowledge about the effectiveness of stimuli-relevant AATP and whether its potential effectiveness can be further enhanced by implementing it in a VR bar. If proven effective, future studies should examine the effect of VR-based AATP in the longer term as well as its cost-effectiveness as adjunctive treatment.

Combining psychological treatments that address AUD-related cognitive dysfunctions in the reflective and impulsive systems might better prepare patients for their inevitable confrontation with alcohol in their natural environment and increase the probability of preventing relapses in the longer term. The stimuli-relevant VR-based AATP can, like the stimuli-relevant conventional AATP, be implemented easily and cheaply as add-on treatment or continued care to enhance the effectiveness of current evidence-based treatment.

## Supplementary information

**Additional file 1.**

## Data Availability

The datasets pertaining to the study are available on request from the authors, provided this is compliant with national legislations and with the decisions of the ethical committees of the respective countries.

## Abbreviations

AASE: Alcohol abstinence self-efficacy scale
AUD: Alcohol use disorder
AAT: Approach avoidance task
AATP: Approach avoidance training program
BDI: Beck depression inventory
BIS: Barratt impulsiveness scale
CBT: Cognitive behavior therapy
CET: Cue exposure therapy
OCDS: Obsessive-compulsive drinking scale
TAU: Treatment as usual
TLFB: Timeline followback
VR: Virtual reality

## Footnotes

1 In addition, patients randomized to the experimental groups will be requested to report their immediate level of cravings on the VAS scale at the beginning and at the end of the AATP session to test for immediate pre- and post-training effects (see Conventional approach- avoidance training program and Approach- avoidance training program in virtual reality sections).

## References

[1] Bates ME, Bowden SC, Barry D (2002). Neurocognitive impairment associated with alcohol use disorders: implications for treatment. Exp Clin Psychopharmacol.

[2] Bates ME, Pawlak AP, Tonigan JS, Buckman JF (2006). Cognitive impairment influences drinking outcome by altering therapeutic mechanisms of change. Psychol Addict Behav.

[3] Bates ME, Buckman JF, Nguyen TT (2013). A role for cognitive rehabilitation in increasing the effectiveness of treatment for alcohol use disorders. Neuropsychol Rev.

[4] LeDoux J. The emotional brain: the mysterious underpinnings of emotional life. New York: Simon and Schuster; 1998.

[5] Strack F, Deutsch R (2004). Reflective and impulsive determinants of social behavior. Pers Soc Psychol Rev.

[6] Bechara A (2005). Decision making, impulse control and loss of willpower to resist drugs: a neurocognitive perspective. Nat Neurosci.

[7] Verdejo-Garcia A, Bechara A (2009). A somatic marker theory of addiction. Neuropharmacology.

[8] Manning V, Staiger PK, Hall K, Garfield JB, Flaks G, Leung D (2016). Cognitive bias modification training during inpatient alcohol detoxification reduces early relapse: a randomized controlled trial. Alcohol Clin Exp Res.

[9] Ernst LH, Plichta MM, Dresler T, Zesewitz AK, Tupak SV, Haeussinger FB (2014). Prefrontal correlates of approach preferences for alcohol stimuli in alcohol dependence. Addict Biol.

[10] Stacy AW, Wiers RW (2010). Implicit cognition and addiction: a tool for explaining paradoxical behavior. Annu Rev Clin Psychol.

[11] Mellentin AI, Skot L, Nielsen B, Schippers GM, Nielsen AS, Stenager E (2017). Cue exposure therapy for the treatment of alcohol use disorders: a meta-analytic review. Clin Psychol Rev.

[12] Lee J-H, Kwon H, Choi J, Yang B-H (2007). Cue-exposure therapy to decrease alcohol craving in virtual environment. Cyberpsychol Behav.

[13] Ghiţă A, Gutiérrez-Maldonado J (2018). Applications of virtual reality in individuals with alcohol misuse: a systematic review. Addict Behav.

[14] Fadardi JS, Cox WM (2009). Reversing the sequence: reducing alcohol consumption by overcoming alcohol attentional bias. Drug Alcohol Depend.

[15] Schoenmakers TM, de Bruin M, Lux IF, Goertz AG, Van Kerkhof DH, Wiers RW (2010). Clinical effectiveness of attentional bias modification training in abstinent alcoholic patients. Drug Alcohol Depend.

[16] Wiers RW, Rinck M, Kordts R, Houben K, Strack F (2010). Retraining automatic action-tendencies to approach alcohol in hazardous drinkers. Addiction (Abingdon, England).

[17] Rinck M, Becker ES (2007). Approach and avoidance in fear of spiders. J Behav Ther Exp Psychiatry.

[18] Wiers RW, Rinck M, Dictus M, van den Wildenberg E (2009). Relatively strong automatic appetitive action-tendencies in male carriers of the OPRM1 G-allele. Genes Brain Behav.

[19] Wiers RW, Eberl C, Rinck M, Becker ES, Lindenmeyer J (2011). Retraining automatic action tendencies changes alcoholic patients’ approach bias for alcohol and improves treatment outcome. Psychol Sci.

[20] Eberl C, Wiers RW, Pawelczack S, Rinck M, Becker ES, Lindenmeyer J (2013). Approach bias modification in alcohol dependence: do clinical effects replicate and for whom does it work best?. Dev Cogn Neurosci.

[21] Wiers CE, Ludwig VU, Gladwin TE, Park SQ, Heinz A, Wiers RW (2015). Effects of cognitive bias modification training on neural signatures of alcohol approach tendencies in male alcohol-dependent patients. Addict Biol.

[22] Rohsenow DJ, Monti PM, Rubonis AV, Gulliver SB, Colby SM, Binkoff JA (2001). Cue exposure with coping skills training and communication skills training for alcohol dependence: 6-and 12-month outcomes. Addiction.

[23] Dawe S, Rees VW, Mattick R, Sitharthan T, Heather N (2002). Efficacy of moderation-oriented cue exposure for problem drinkers: a randomized controlled trial. J Consult Clin Psychol.

[24] Loeber S, Croissant B, Heinz A, Mann K, Flor H (2006). Cue exposure in the treatment of alcohol dependence: effects on drinking outcome, craving and self-efficacy. Br J Clin Psychol.

[25] Wiers CE, Stelzel C, Park SQ, Gawron CK, Ludwig VU, Gutwinski S (2014). Neural correlates of alcohol-approach bias in alcohol addiction: the spirit is willing but the flesh is weak for spirits. Neuropsychopharmacology.

[26] Wiers RW, Houben K, Fadardi JS, Van Beek P, Rhemtulla M, Cox WM (2015). Alcohol cognitive bias modification training for problem drinkers over the web. Addict Behav.

[27] Loijen A, Rinck M, Walvoort SJW, Kessels RPC, Becker ES, Egger JIM (2018). Modification of automatic alcohol-approach tendencies in alcohol-dependent patients with mild or major neurocognitive disorder. Alcohol Clin Exp Res.

[28] Wiers CE, Stelzel C, Gladwin TE, Park SQ, Pawelczack S, Gawron CK (2015). Effects of cognitive bias modification training on neural alcohol cue reactivity in alcohol dependence. Am J Psychiatry.

[29] Chan A-W, Tetzlaff JM, Altman DG, Laupacis A, Gøtzsche PC, Krleža-Jerić K (2013). SPIRIT 2013 statement: defining standard protocol items for clinical trials. Ann Intern Med.

[30] Schulz KF, Altman DG, Moher D (2010). CONSORT 2010 statement: updated guidelines for reporting parallel group randomised trials. BMC Med.

[31] Health N. C. C. f. M., Health N. I. f., Excellence C. Alcohol use disorders: the NICE guideline on the diagnosis, assessment and management of harmful drinking and alcohol dependence. New York: RCPsych Publications; 2011.

[32] Eberl C, Wiers RW, Pawelczack S, Rinck M, Becker ES, Lindenmeyer JJAC (2014). Implementation of approach bias re-training in alcoholism—how many sessions are needed?. Alcohol Clin Exp Res.

[33] Supplementary Material 2020.

[34] Sheehan DV, Lecrubier Y, Sheehan KH, Amorim P, Janavs J, Weiller E (1998). The Mini-International Neuropsychiatric Interview (MINI): the development and validation of a structured diagnostic psychiatric interview for DSM-IV and ICD-10.

[35] Sheehan DV (2015). Mini International Neuropsychiatric Interview 7.0.

[36] McLellan AT, Kushner H, Metzger D, Peters R, Smith I, Grissom G (1992). The fifth edition of the addiction severity index. J Subst Abus Treat.

[37] Kokkevi A, Hartgers C (1995). EuropASI: European adaptation of a multidimensional assessment instrument for drug and alcohol dependence. Eur Addict Res.

[38] Sobell MB, Maisto S, Sobell LC, Cooper A, Cooper T, Sanders B (1980). Developing a prototype for evaluating alcohol treatment effectiveness.

[39] Sobell LC, Brown J, Leo GI, Sobell MB (1996). The reliability of the alcohol timeline followback when administered by telephone and by computer. Drug Alcohol Depend.

[40] Wewers ME, Lowe NK (1990). A critical review of visual analogue scales in the measurement of clinical phenomena. Res Nurs Health.

[41] Drobes DJ, Thomas SE (1999). Assessing craving for alcohol. Alcohol Res.

[42] Anton RF (2000). Obsessive–compulsive aspects of craving: development of the Obsessive Compulsive Drinking Scale. Addiction.

[43] DiClemente CC, Carbonari JP, Montgomery RP, Hughes SO (1994). The alcohol abstinence self-efficacy scale. J Stud Alcohol.

[44] Patton JH, Stanford MS, Barratt ES (1995). Factor structure of the Barratt impulsiveness scale. J Clin Psychol.

[45] Beck AT, Steer RA, Ball R, Ranieri WF (1996). Comparison of Beck Depression Inventories-IA and-II in psychiatric outpatients. J Pers Assess.

[46] Gordon B, Caramazza A (1982). Lexical decision for open-and closed-class words: failure to replicate differential frequency sensitivity. Brain Lang.

[47] Gordon B (1983). Lexical access and lexical decision: mechanisms of frequency sensitivity. J Verbal Learn Verbal Behav.

[48] Vickers AJ, Altman DG (2001). Analysing controlled trials with baseline and follow up measurements. BMJ.

[49] Van Buuren S. Flexible imputation of missing data. New York: CRC Press; 2018.

[50] Borenstein M, Rothstein H, Cohen J, Schoefeld D, Berlin J, Lakatos E (2001). Power and precision TM.

[51] Faul F, Erdfelder E, Lang A-G, Buchner A (2007). G* Power 3: a flexible statistical power analysis program for the social, behavioral, and biomedical sciences. Behav Res Methods.

[52] Phaf RH, Mohr SE, Rotteveel M, Wicherts JM (2014). Approach, avoidance, and affect: a meta-analysis of approach-avoidance tendencies in manual reaction time tasks. Front Psychol.

[53] Kersbergen I, Woud ML, Field M (2015). The validity of different measures of automatic alcohol action tendencies. Psychol Addict Behav.

[54] Wittekind CE, Reibert E, Takano K, Ehring T, Pogarell O, Rüther T (2019). Approach-avoidance modification as an add-on in smoking cessation: a randomized-controlled study. Behav Res Ther.

[55] Machulska A, Zlomuzica A, Rinck M, Assion HJ, Margraf J (2016). Approach bias modification in inpatient psychiatric smokers. J Psychiatr Res.

[56] Sherman BJ, Baker NL, Squeglia LM, McRae-Clark AL (2018). Approach bias modification for cannabis use disorder: a proof-of-principle study. J Subst Abus Treat.

[57] Carter BL, Tiffany ST (1999). Meta-analysis of cue-reactivity in addiction research. Addiction (Abingdon, England).

[58] Sinha R, O’Malley SS (1999). Craving for alcohol: findings from the clinic and the laboratory. Alcohol Alcohol (Oxford, Oxfordshire).

[59] Sinha R, Li CS (2007). Imaging stress- and cue-induced drug and alcohol craving: association with relapse and clinical implications. Drug Alcohol Rev.

[60] Kuntze MF, Stoermer R, Mager R, Roessler A, Mueller-Spahn F, Bullinger AH (2001). Immersive virtual environments in cue exposure. Cyberpsychol Behav.

[61] Stasiewicz PR, Brandon TH, Bradizza CM (2007). Effects of extinction context and retrieval cues on renewal of alcohol-cue reactivity among alcohol-dependent outpatients. Psychol Addict Behav.

[62] Fuchs R, Lasseter H, Ramirez D, Xie X (2008). Relapse to drug seeking following prolonged abstinence: the role of environmental stimuli. Drug Discov Today Dis Model.

[63] Bordnick PS, Traylor A, Copp HL, Graap KM, Carter B, Ferrer M (2008). Assessing reactivity to virtual reality alcohol based cues. Addict Behav.

[64] Cho S, Ku J, Park J, Han K, Lee H, Choi YK (2008). Development and verification of an alcohol craving-induction tool using virtual reality: craving characteristics in social pressure situation. Cyberpsychol Behav.

[65] Lee JS, Namkoong K, Ku J, Cho S, Park JY, Choi YK (2008). Social pressure-induced craving in patients with alcohol dependence: application of virtual reality to coping skill training. Psychiatry Investig.

[66] Ryan JJ, Kreiner DS, Chapman MD, Stark-Wroblewski K (2010). Virtual reality cues for binge drinking in college students. Cyberpsychol Behav Soc Netw.

[67] Kim DY, Lee JH (2015). Development of a virtual approach-avoidance task to assess alcohol cravings. Cyberpsychol Behav Soc Netw.

[68] Ferrer-García M, García-Rodríguez O, Gutiérrez-Maldonado J, Pericot-Valverde I, Secades-Villa R (2010). Efficacy of virtual reality in triggering the craving to smoke: its relation to level of presence and nicotine dependence. Stud Health Technol Inform.

[69] García-Rodríguez O, Weidberg S, Gutiérrez-Maldonado J, Secades-Villa R (2013). Smoking a virtual cigarette increases craving among smokers. Addict Behav.

[70] Lee JH, Ku J, Kim K, Kim B, Kim IY, Yang BH (2003). Experimental application of virtual reality for nicotine craving through cue exposure. Cyberpsychol Behav.

[71] Kwon H, Choi J, Roh S, Yang B, Lee J (2006). Application of virtual reality-cue exposure therapy for reducing alcohol craving. Annu Rev Cyberther Telemed.

[72] Lee SH, Han DH, Oh S, Lyoo IK, Lee YS, Renshaw PF (2009). Quantitative electroencephalographic (qEEG) correlates of craving during virtual reality therapy in alcohol-dependent patients. Pharmacol Biochem Behav.

[73] Son JH, Lee SH, Seok JW, Kee BS, Lee HW, Kim HJ (2015). Virtual reality therapy for the treatment of alcohol dependence: a preliminary investigation with positron emission tomography/computerized tomography. J Stud Alcohol Drugs.

[74] Field M, Cox WM (2008). Attentional bias in addictive behaviors: a review of its development, causes, and consequences. Drug Alcohol Depend.

[75] Field M, Munafo MR, Franken IH (2009). A meta-analytic investigation of the relationship between attentional bias and subjective craving in substance abuse. Psychol Bull.

[76] Brockmeyer T, Hahn C, Reetz C, Schmidt U, Friederich H-C (2015). Approach bias and cue reactivity towards food in people with high versus low levels of food craving. Appetite.

[77] Lender A, Meule A, Rinck M, Brockmeyer T, Blechert J (2018). Measurement of food-related approach–avoidance biases: larger biases when food stimuli are task relevant. Appetite.

[78] Van Dessel P, De Houwer J, Gast A (2016). Approach–avoidance training effects are moderated by awareness of stimulus–action contingencies. Personal Soc Psychol Bull.

[79] Lavie N (1995). Perceptual load as a necessary condition for selective attention. J Exp Psychol Hum Percept Perform.

[80] Nishiguchi Y, Takano K, Tanno Y (2015). Explicitly guided attentional bias modification promotes attentional disengagement from negative stimuli. Emotion.

[81] Krebs G, Hirsch CR, Mathews A (2010). The effect of attention modification with explicit vs. minimal instructions on worry. Behav Res Ther.

[82] Grafton B, Mackintosh B, Vujic T, MacLeod C (2014). When ignorance is bliss: explicit instruction and the efficacy of CBM-A for anxiety. Cogn Ther Res.

