# Supplementary material for: A randomized controlled trial of a virtual reality based, approach-avoidance training program for alcohol use disorder: a study protocol
Source: BMC Psychiatry. 2020 Jun 30;20:340. doi: 10.1186/s12888-020-02739-1 (PMC7324964; doi:10.1186/s12888-020-02739-1)
Supplement: Supplementary file 1 — Additional file 1. [file 12888_2020_2739_MOESM1_ESM.zip › 03_06_20_Supplementary materialR2.docx]

**Supplementary Material**

1. **Stimuli for the stimulus-irrelevant and stimulus-relevant diagnostic AATs and for the AATP**

The stimuli for the diagnostic AATs are the same for the PC- and VR-AATs and are used in both the stimulus-relevant and stimuli-irrelevant version. There are a total of N = 28 high-poly objects, of which 14 are non-alcoholic and 14 are alcoholic beverages. In software development, the term ‘high-poly’ refers to 3D computer models consisting of a high number of polygons, which makes their overall quality (resolution) much higher and allows for a more realistic and detailed look (compared to low-poly 3D objects). The stimulus set in the diagnostic AATs consists of 14 stimuli (7 alcoholic, 7 non-alcoholic) taken from the AAT-training stimuli set (training effect for known stimuli), while the other 14 stimuli (7 alcoholic, 7 non-alcoholic) are exclusively presented in the diagnostic AATs (transfer to unknown stimuli). Examples of alcoholic and non-alcoholic beverages exclusively displayed in the diagnostic AATs are shown in Supplementary Figure 1. The stimuli used for both the VR and PC AAT-training are also high-poly objects, and the total number of stimuli is N = 50. For further details on the experiments, including stimulus presentation information, please refer to the next sections.

Insert Figure 1. around here

**Figure 1.** Examples of stimuli from the diagnostic AATs picture set

1. **Stimulus irrelevant and stimulus-relevant diagnostic AATs**

Both a non-stimulus-relevant and a stimulus-relevant diagnostic AAT is run with the same 28 stimuli (14 alcoholic, 14 non-alcoholic). The order of the diagnostic tests is randomized. Before the actual diagnostic AAT starts, participants practice the movements in ten trials with beer vs. water stimuli, which are not part of the actual diagnostic AAT stimuli set. During practice, feedback is given in case mistakes are made (auditory signal and red light in VR; red ‘ERROR’ message on the PC).

In the stimulus-irrelevant (implicit) AAT, participants are either instructed to respond to a yellow frame with a pull movement and a blue frame with a push movement or the other way around. Participants are randomly assigned to one of the color conditions. On the PC, the responses are implemented with the joystick, which participants are instructed to fully extend/retract. To provide visual feedback for an approach (pull) movement, the images are zoomed in/ become larger vs. zoomed out/ become smaller for an avoidance (push) movement. In the VR, the bartender places the beverage on top of the bar in front of the participant. The moment the beverage is placed on the bar counter, a colored frame (yellow or blue) instantly appears around the object. After the practice trials, each of the 28 stimuli are presented in randomized order. Each stimulus is presented twice, once in a push-condition and once in a pull-condition, resulting in 56 trials in total. The VR controller is visible in the application and acts like a hand replacement. Participants need to use their arm to draw the beverage towards themselves whilst pushing a lever on the back side of the controller with their index finger to grasp the stimuli and pull it into a slot in front of them. To push a beverage away, the arm needs to be extended and the controller has to hit the stimuli so it drops and disappears in a slot at the other side of the bar counter. This is supported by auditory feedback of a crashing bottle. For an impression of the VR, please refer to Figure 2.

Insert Figure 2. around here

**Figure 2.** Bartender placing an alcoholic beverage in front of the participant at the virtual bar.

As stated above, the stimuli for the explicit AAT are the same as for the implicit AAT and are used in both the PC- and VR-version. The stimulus-relevant AAT also consists of two blocks in randomized order. In block A, participants are explicitly instructed to react to alcoholic stimuli with a pull movement and to non-alcoholic beverages with a push movement. In block B, the instructions are reversed (i.e. push alcohol, pull soft-drinks). The total number of trials is 56, whereby each beverage is pulled once and pushed once, depending on the block condition.

As in the implicit AATs, the responses are implemented with a joystick on the PC. To provide visual feedback for the participant, the images zoom in/ become larger when pulled towards oneself vs. zoom out/ become smaller with a push reaction. In the VR, the bartender places the beverage in front of the participant who needs to carry out the push/pull movement (as described above) according to the type of beverage (alcoholic vs. non-alcoholic) and depending on the instructions (block A vs. block B). The total duration of each AAT (implicit and explicit) in VR and on the PC were piloted and subsequently estimated to take between 6-10 minutes, depending on how fast the individual participants respond.

1. **Approach Avoidance Training in VR vs. PC**

The stimuli used for the PC-based and VR-based training are identical. The training is explicit, i.e. participants are directly instructed by the experimenter to pull non-alcoholic drinks towards themselves and push alcoholic beverages away. To get acquainted with the VR-movements, participants have the opportunity to practice individually in a practice mode with a beer and a water stimulus until they are familiar with the exact movements required in the VR environment. Since the joystick movements in the PC version are easier to execute, there is a standard of 10 practice trials, but in case of obvious difficulties, the practice mode can be repeated. For standardization, the training is constrained to three repetitions per stimulus, resulting in a total of 150 trials. The training was piloted and is estimated to take about 20-30 minutes per session, depending on movement familiarity.

1. **Response Inhibition: GoNogo task**

14 stimuli (7 alcoholic, 7 non-alcoholic) from the AAT-training high-poly picture set (different ones than used in the diagnostic AATs) plus 14 novel photo stimuli (7 depicting alcoholic and 7 depicting non-alcoholic beverages; examples are shown in Figure 3) are included in the GoNoGo task. The overlapping stimuli from the training set were included to cover training effects and the novel stimuli were included to capture transfer/ generalization effects. In the GoNoGo task, the SOA (stimulus onset asynchrony) is randomly varied (with replacement) with durations between 1000 and 3000ms (100ms increments). Participants are instructed to respond to non-alcoholic beverages as fast as possible, pressing the space bar on the keyboard (= ‘go’ trial). They are instructed not to respond when an alcoholic stimulus is presented (= ‘no-go’ trial). There are 70 trials in total in randomized order: 14 trials are no-go trials and 56 are go trials. Participants inhibit responses to each of the alcoholic stimuli, which are only presented once (7 high-poly, trained; 7 photos, novel), while the non-alcoholic beverages are shown four times. Each image is presented for 1500ms. Participants receive immediate feedback if they make a mistake by a red cross appearing on the screen (which they are informed about in the instructions beforehand). Before the actual task starts, there is a practice block with two standard stimuli (beer vs. water). There is a maximum error rate of 10% within the exercise cycle. If this error rate is exceeded, the practice block is repeated.

Insert Figure 3. around here

**Figure 3.** Examples of the photo stimuli used in the GoNogo task.
