# Supplementary figures and images for: A randomized controlled trial of a virtual reality based, approach-avoidance training program for alcohol use disorder: a study protocol
Source: BMC Psychiatry. 2020 Jun 30;20:340. doi: 10.1186/s12888-020-02739-1 (PMC7324964; doi:10.1186/s12888-020-02739-1)

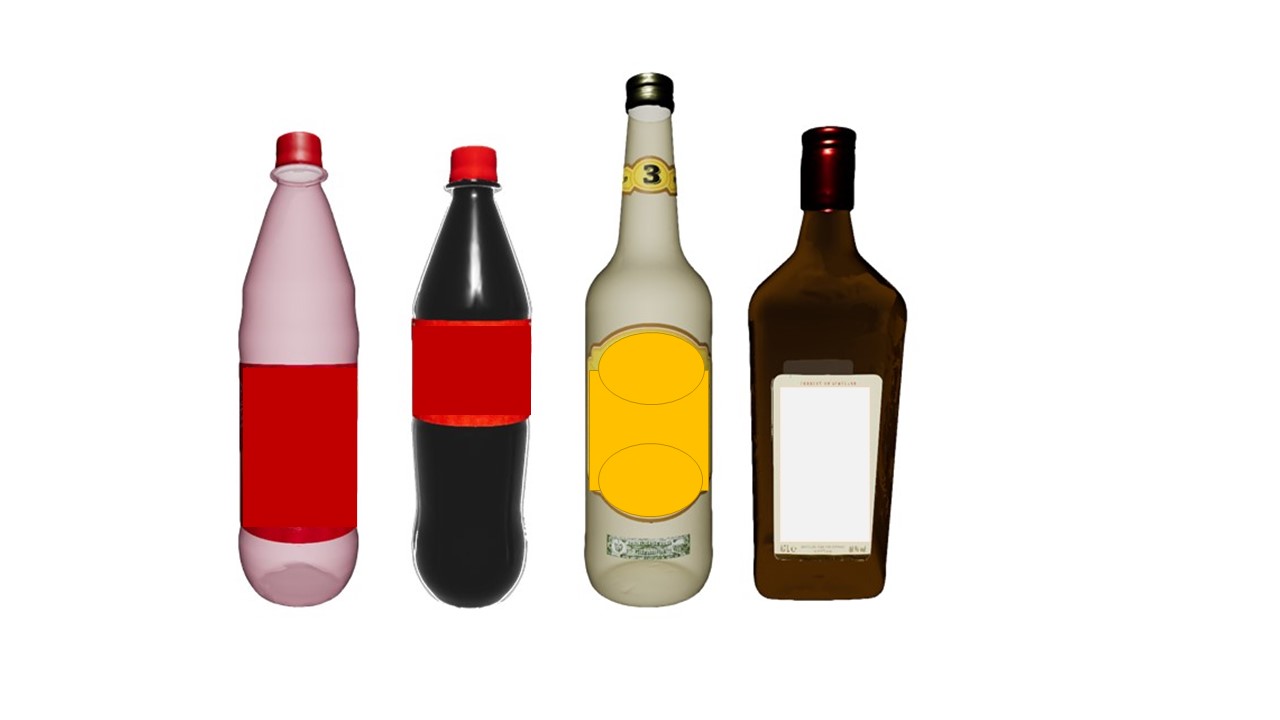

Supplement: Supplementary file 1 — Additional file 1. [file 12888_2020_2739_MOESM1_ESM.zip › 03_06_20_Figure 1._Supplementary_MaterialsR2.jpg]

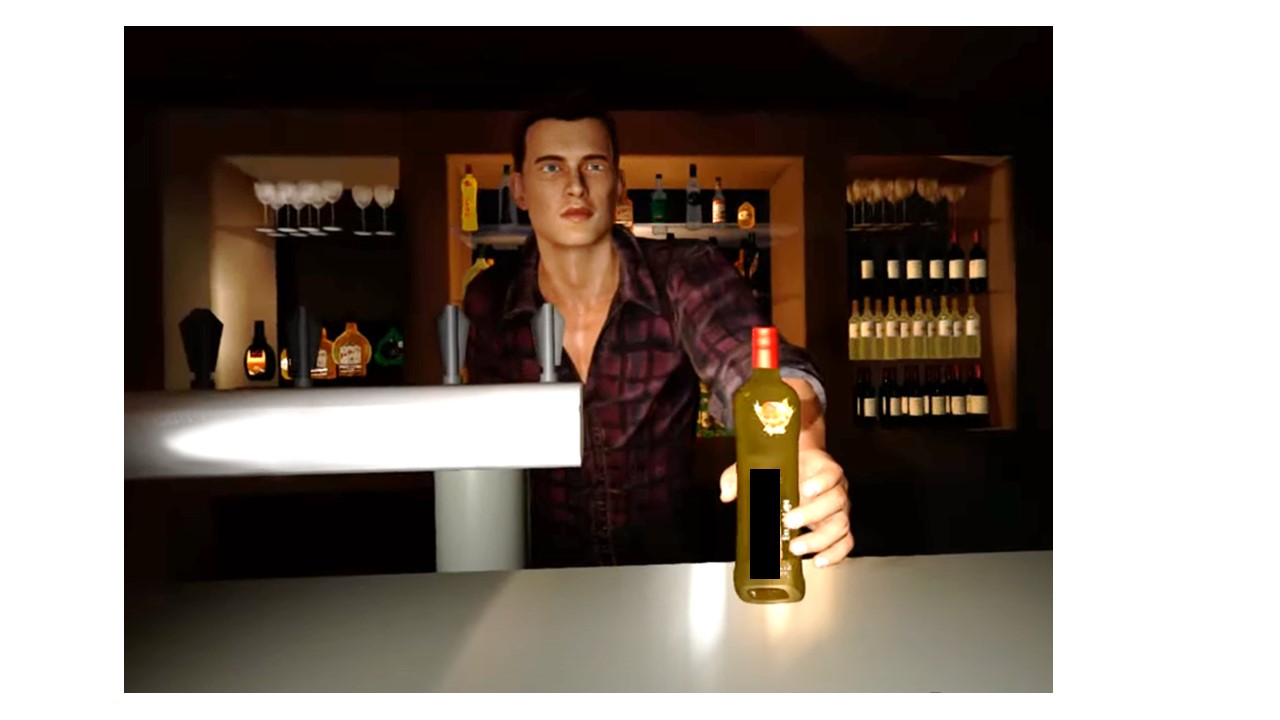

Supplement: Supplementary file 1 — Additional file 1. [file 12888_2020_2739_MOESM1_ESM.zip › 03_06_20_Figure 2._Supplementary_Materials. R2.jpg]

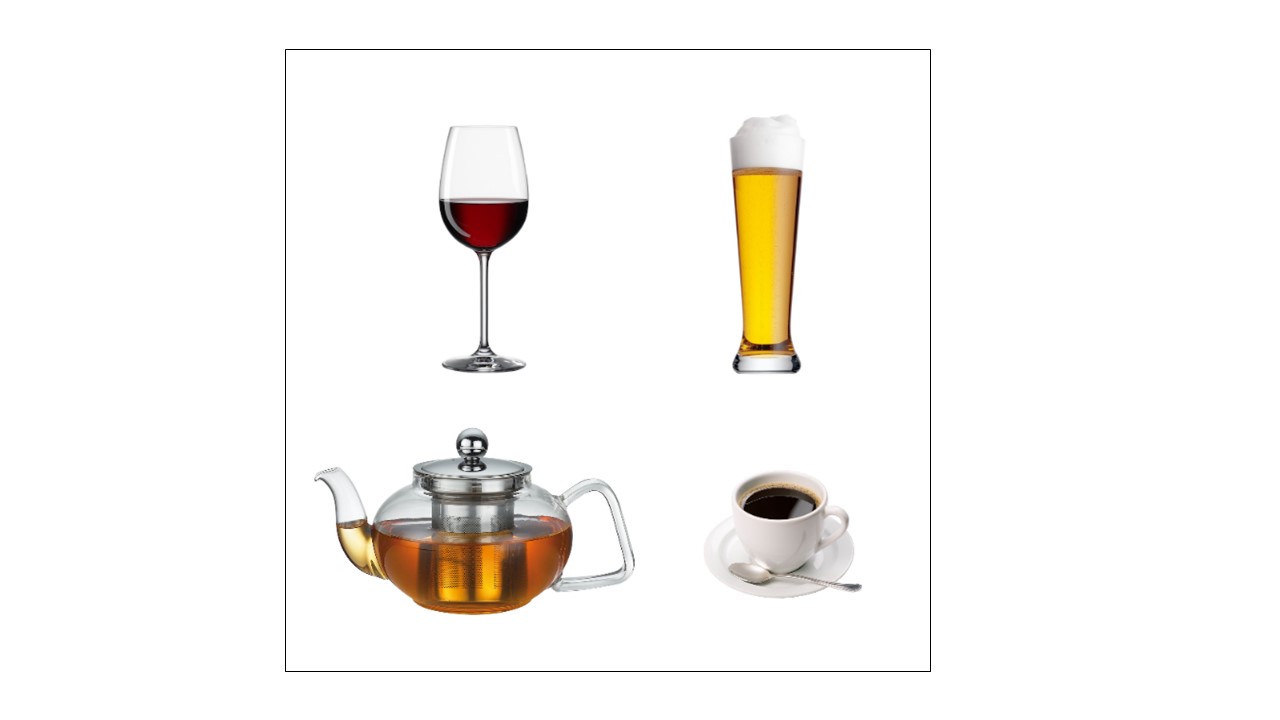

Supplement: Supplementary file 1 — Additional file 1. [file 12888_2020_2739_MOESM1_ESM.zip › 03_06_20_Figure 3._Supplementary_Materials.R2.jpg]
